# Genome-centric metagenomics reveals insights into the evolution and metabolism of a new free-living group in Rhizobiales

**DOI:** 10.1186/s12866-021-02354-4

**Published:** 2021-10-28

**Authors:** Leandro Nascimento Lemos, Fabíola Marques de Carvalho, Alexandra Gerber, Ana Paula C. Guimarães, Celio Roberto Jonck, Luciane Prioli Ciapina, Ana Tereza Ribeiro de Vasconcelos

**Affiliations:** 1 Bioinformatics Laboratory, National Laboratory of Scientific Computing (LNCC), Av. Getúlio Vargas, 333 - Quitandinha, Petrópolis, RJ 25651-076 Brazil; 2 Leopoldo Americo Miguez de Melo Research Center (CENPES-Petrobras), Rio de Janeiro, RJ Brazil

**Keywords:** Rhizobiales, Integration of genomic public data, Aestuariivirgaceae, Evolution, Uncultivated lineages

## Abstract

**Background:**

The Rhizobiales (Proteobacteria) order is an abundant and diverse group of microorganisms, being extensively studied for its lifestyle based on the association with plants, animals, and humans. New studies have demonstrated that the last common ancestor (LCA) of Rhizobiales had a free-living lifestyle, but the phylogenetic and metabolism characterization of basal lineages remains unclear. Here, we used a high-resolution phylogenomic approach to test the monophyly of the Aestuariivirgaceae family, a new taxonomic group of Rhizobiales. Furthermore, a deep metabolic investigation provided an overview of the main functional traits that can be associated with its lifestyle. We hypothesized that the presence of pathways (e.g., Glycolysis/Gluconeogenesis) and the absence of pathogenic genes would be associated with a free-living lifestyle in Aestuariivirgaceae.

**Results:**

Using high-resolution phylogenomics approaches, our results revealed a clear separation of Aestuariivirgaceae into a distinct clade of other Rhizobiales family, suggesting a basal split early group and corroborate the monophyly of this group. A deep functional annotation indicated a metabolic versatility, which includes putative genes related to sugar degradation and aerobic respiration. Furthermore, many of these traits could reflect a basal metabolism and adaptations of Rhizobiales, as such the presence of Glycolysis/Gluconeogenesis pathway and the absence of pathogenicity genes, suggesting a free-living lifestyle in the Aestuariivirgaceae members.

**Conclusions:**

Aestuariivirgaceae (Rhizobiales) family is a monophyletic taxon of the Rhizobiales with a free-living lifestyle and a versatile metabolism that allows these microorganisms to survive in the most diverse microbiomes, demonstrating their adaptability to living in systems with different conditions, such as extremely cold environments to tropical rivers.

**Supplementary Information:**

The online version contains supplementary material available at 10.1186/s12866-021-02354-4.

## Background

The Rhizobiales (Proteobacteria) order is abundant, diverse and widespread in several environments [1]. Due to their association with plant, animal, and human diseases, and their economic impact, many microorganisms of this group have been widely studied applying molecular biology technologies (metagenomics, ARISA/T-RFLP, geochips, 16S rRNA sequencing). In plants, Rhizobiales order includes symbionts that establish mutualistic and pathogenic relationships. *Rhizobium, Bradyrhizobium, Azorhizobium* and others genera form a symbiotic association with legumes and are responsible for the nitrogen fixation process (for a review see [2]) while *Agrobacterium* belongs to the pathogenic group [3]. Members of the Rhizobiales order have been found in association with lichens [4], as a third member of this ecological relationship. The genera *Brucella* and *Bartonella* are associated with animal and human diseases [5]. In marine environments, Rhizobiales have been associated with diseases in corals [6], however, it has not been proven as the causative agent and could be only an opportunistic bacteria identified in diseased tissues**.** In water column microbiomes surrounding the giant kelp *Macrocystis pyrifera*, Rhizobiales abundance was associated with an increased carbon dioxide (pCO2) [7]. Ng and Chiu [8] observed that an increase in Rhizobiales may be associated with the increase of nutrients that lead to hypoxia and acidification of the oceans.

To date (August 2021), 6983 Rhizobiales genomes are available in the Genome Taxonomy Database (GTDB – [9]), which include nitrogen-fixing plant symbionts (*Rhizobium* and *Bradyrhizobium*), plant and human pathogens (Candidatus *Liberibacter* and *Brucella*) or free-living in soil (*Methylobacterium*). However, some of these genomes deposited in public repositories represent new taxonomic groups and have not been individually explored in the evolutionary and metabolic context. To complement microbiological studies and highlight new discoveries of evolution and metabolism of new taxonomic groups, the reconstruction of genomes from metagenomes samples has been applied in several microbiome datasets [10–13]. Briefly, metagenomic reads were assembled into contigs and then contigs were clustered into individual populations, where each population represents a potential microbial genome [14]. The main advantage of this approach is to access taxonomic and metabolic information of microorganism groups that lack cultivated reference genomes. This includes the description of new archaeal and bacterial lineages [12] and their roles in several microbiomes. Recent advances in assembly and binning algorithms have provided accurate and biological validations predicted in silico results of taxonomic groups discovered by reconstruction of genomes from metagenomes, which were later cultivated and validated by the use of cultivation methods [15].

New taxa have been affiliated to the order Rhizobiales, which include the Aestuariivirgaceae (Rhizobiales) family proposed by [16] during the description and whole-genome-sequence of the *Aestuariivirga litoralis* species. This group was first described as part of an investigation to understand estuarine sediments’ microbiome, highlighting significant phenotypic and genomic characterization findings. Furthermore, initial phylogeny analysis based on 16S rRNA and protein marker genes showed that his group should represent a new family [16]. However, an investigation using additional genomes is necessary to corroborate the monophyly of this group, once its phylogenetic position remains unclear. Besides, a deep metabolic investigation can provide new insights into the functional traits and lifestyle of Aestuariivirgaceae in terrestrial and water environments.

In this study we used Metagenome-Assembled Genomes (MAGs) and whole-genome-sequenced bacterial isolates to test the monophyly and to describe metabolic profile of the Aestuariivirgaceae family that can be associated with its lifestyle. We hypothesized that the presence of pathways (e.g., Glycolysis/Gluconeogenesis) and the absence of pathogenic genes would be associated with a free-living lifestyle in Aestuariivirgaceae.

## Results and discussion

To test the monophyly and to predict the putative central metabolism of the Aestuariivirgaceae (Rhizobiales) family, we used a dataset with 19 whole-genome sequenced bacterial isolates and Metagenome-Assembled Genomes (MAGs) (Table 1). Firstly, we reconstructed a new metagenome-assembled genome (MAG - named METAPETRO_BR_BIN_54) using marine sediment metagenomes (Supplementary Table 1). Specifically, METAPETRO_BR_BIN_54 has 93.7% of completeness and 2.17% of contamination (Table 1). According to Minimum information about a metagenome-assembled genome of bacteria and archaea (MIMAG) standards [14] and CheckM classification [17], MAGs with more than 90% of completeness and less than 5% of contamination are considered high-quality and near-complete genomes. We reinforce that 2.17 represents genomes with lower percentages of contamination. To complete these analyses, we also add 18 genomes [11, 12, 16, 18–23] deposited in public sequence repositories (Table 1), which were not explored deeply in the context of this investigation. Also according to MIMAG standards [14], these genomes were assigned with high-quality or medium-quality drafts (Table 1). We found Aestuariivirgaceae members in a broad of several environments (Table 1), such as terrestrial (soil, permeable sediments, and phosphatic stromatolites formations) and aquatic (marine sediments, artificial well, wastewater treatment plant, High Arctic freshwater, and Amazon Basin River), demonstrating their adaptability to living in systems with different conditions, such as extremely cold environments to tropical rivers.

**Table 1:** Genomic features of Aestuariivirgaceae (Rhizibiales; Proteobacteria) genomes isolated or reconstructed using metagenomes

Genome	Number of contigs	Size (Mbp)	Environment	Taxonomy (GTDB)	Respiration	Completeness/Contamination	Genome Accession	Reference
*Aestuariivirga litoralis*	26	4.2	Water	g__Aestuariivirga; s__Aestuariivirga litoralis	Aerobic	98.5/0.4	GCF_003234965.1	[16]
Palsa_927	294	2.59	Palsa	g__Aestuariivirga; s__Aestuariivirga sp003151375	Aerobic	82.2/2.4	GCA_003151375.1	[12]
METAPETRO_BR_BIN_54	756	5.06	Marine sediment	g__JABDJG01;s__	Aerobic	93.7/2.17	This study	This study.
SCPDY	37	7.1	Storage Tank/Water	g__Nordella; s__Nordella sp005502925	Aerobic	98.9/0.6	GCF_005502925.1	[11]
X2C	106	7.1	Artificial well/Hydra/Water	s__Nordella sp005502925	Aerobic	100.0/0.2	GCF_005502975.1	[11].
X1A	48	7.1	Artificial well/Hydra/Water	g__Nordella; s__Nordella sp005502925	Aerobic	98.9/0.6	GCF_005502345.1	[11]
AP_21	745	3.8	Soil	g__Nordella; s__Nordella sp005884715	Aerobic	82.68/0.69	GCA_005884715.1	[12]
Bin_29_15	222	3.2	High Arctic freshwater	g__Aestuariivirga; s__Aestuariivirga sp009885825	Aerobic	95.79/1.64	GCA_009885825.1	[19]
ES-bin-180	684	2.8	Nearby exposed soil of glacier	g__Aestuariivirga; s__Aestuariivirga sp014380505	Aerobic	66.54/1.72	GCA_014380505.1	[20]
RU_4_15	370	3.0	Phosphatic stromatolites formations	g__Aestuariivirga; s__Aestuariivirga sp012032065	Aerobic	72.2/2.48	GCA_012032065.1	[21]
SS_bin_17	495	4.3	Permeable (sandy) sediments	g__JABDJG01; s__JABDJG01 sp013002595	Aerobic	89.03/1.20	GCA_013002595.1	[22]
AM_0226	58	2.9	Amazon Basin river	g__Aestuariivirga; s__Aestuariivirga sp900298995	Aerobic	98.79/0.34	GCA_900298995.1	[18]
Loclat_bin-06399	820	3.9	Water Lake	g__Aestuariivirga; s__Aestuariivirga sp903930095	Aerobic	88.97/4.26	GCA_903930095.1	[47]
Loc080925-5m_bin-0050	321	2.7	Water Lake	g__CABJBCQ01; s__CABJBCQ01 sp903951595	Aerobic	95.0/1.96	GCA_903944365.1	[47]
Loc080925–4m_bin-0358	264	2.7	Water Lake	g__CABJBCQ01; s__CABJBCQ01 sp903951595	Aerobic	99.13/3.26	GCA_903951595.1	[47]
Loc080925–4-5m_bin-0281	264	2.7	Water Lake	g__CABJBCQ01; s__CABJBCQ01 sp903951595	Aerobic	99.13/3.26	GCA_903958745.1	[47]
RBC017	344	2.9	Wastewater treatment plant	g__Aestuariivirga; s__Aestuariivirga sp902826365	Aerobic	88.08/0.6	GCA_902826365.1	[23]
RBC019	527	2.8	Wastewater treatment plant	g__Aestuariivirga; Aestuariivirga sp902826365	Aerobic	82.96/3.06	GCA_902826675.1	[23]
RBC065	393	2.7	Wastewater treatment plant	g__Aestuariivirga; Aestuariivirga sp902826365	Aerobic	79.98/1.3	GCA_902826905.1	[23]

**Fig. 1:**
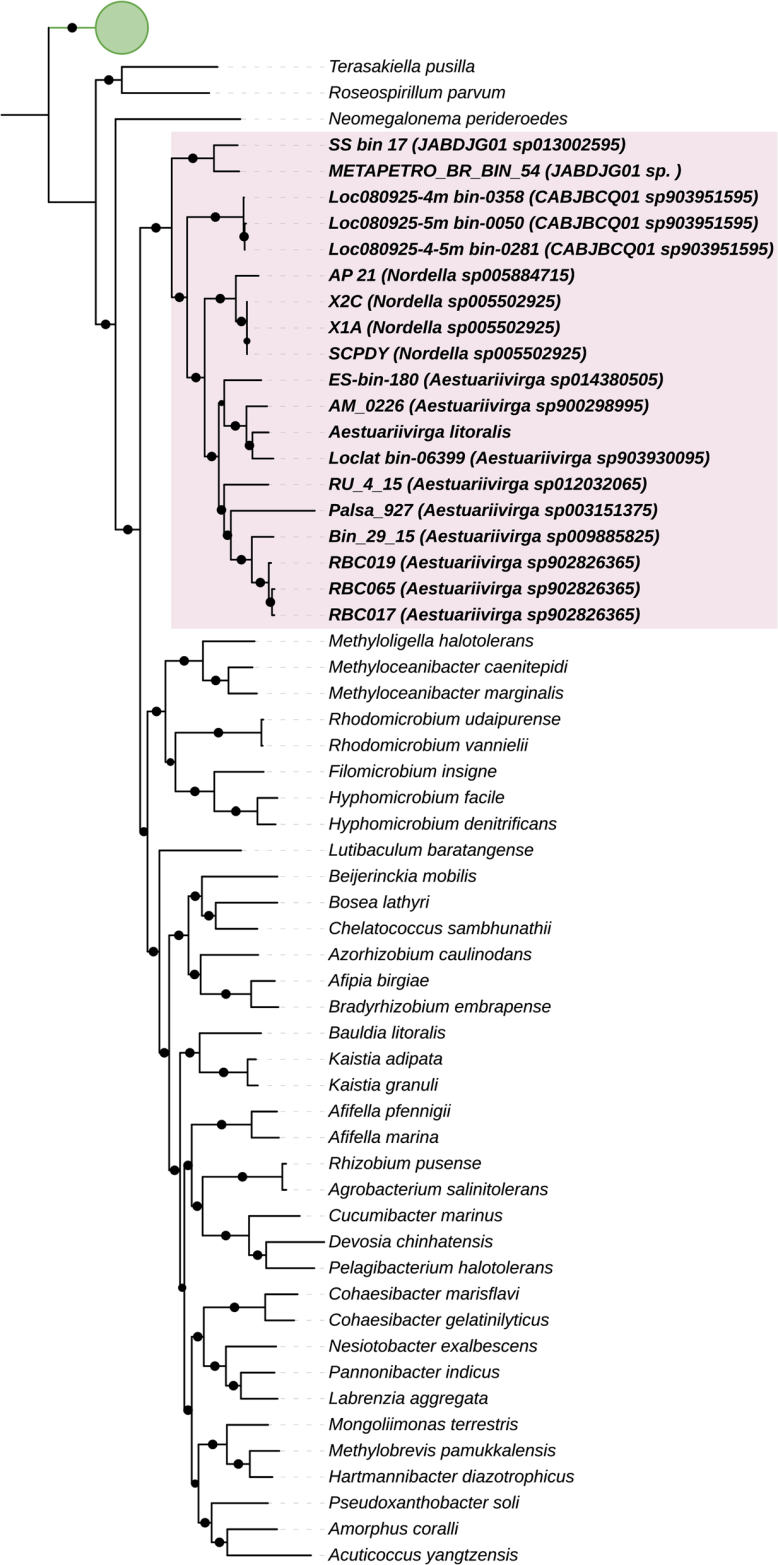
Phylogenomic tree showing the evolutionary position of the Aestuariivirgaceae (Proteobacteria, Rhizobiales) species. The phylogenomic tree was inferred using the alignment and the concatenation of bacterial single-copy core genes (SCGs) (Supplementary Table 3) [39, 40] under the Jones-Taylor-Thorton model and CAT approximation with 20 rate categories. The Aestuariivirgaceae genomes studied here are assigned with a pink color. The nodes that showed a bootstrap support ≥70% are assigned with a black point in the tree. Green circle indicates the outgroup used in the phylogenomic analysis

From 19 genomes, a total of 13 unique species were identified, which includes *Aestuariivirga litoralis* described by Li and collaborators [16]. High-resolution taxonomy prediction based on the rank-normalized GTDB taxonomy with the criteria of relative evolutionary divergence (RED) and ANI indicated the presence of 8 unique species of the genera *Aestuariivirga* (*Aestuariivirga litoralis*, *Aestuariivirga sp902826365*, *Aestuariivirga sp003151375*, *Aestuariivirga sp009885825*, *Aestuariivirga sp012032065*, *Aestuariivirga sp014380505*, *Aestuariivirga sp900298995*, and *Aestuariivirga sp903930095*). *Nordella* genus was represented by two unique species (*Nordella* sp005502925 and *Nordella* sp005884715). This species was identified for the first time using 16S rRNA gene sequence analysis in an ecological interaction with an amoeba from a water tank [24]. We also identified genomes assigned with the genus *JABDJG01* (*JABDJG01 sp013002595* and *JABDJG01 sp*.) and *CABJBCQ01*. Both genera have not been described in previous studies and the taxonomy name reflects the proposal used by the Genome Taxonomy Database. To clarify the phylogenetic position and to test the monophyly of the Aestuariivirgaceae, we used a high-resolution phylogenomic approach based on the alignment and concatenation of single-copy marker genes (Fig. 1). Our results revealed a clear separation of Aestuariivirgaceae family into a distinct clade of other Rhizobiales families (Bootstrap ≳ 95%), indicating that it could seem to be a basal group and may have split early. The formation of this clade validates the monophylic origin of the Aestuariivirgaceae family, which was proposed by Li and collaborators [16]. Our phylogenetic results were the same that predicted by GTDBTk to estimate the taxonomy assignment (Table 1), where *Aestuariivirga sp902826365*, *Nordella sp005502925*, and *CABJBCQ01 sp903951595* were represented by more than one genome.

**Fig. 2:**
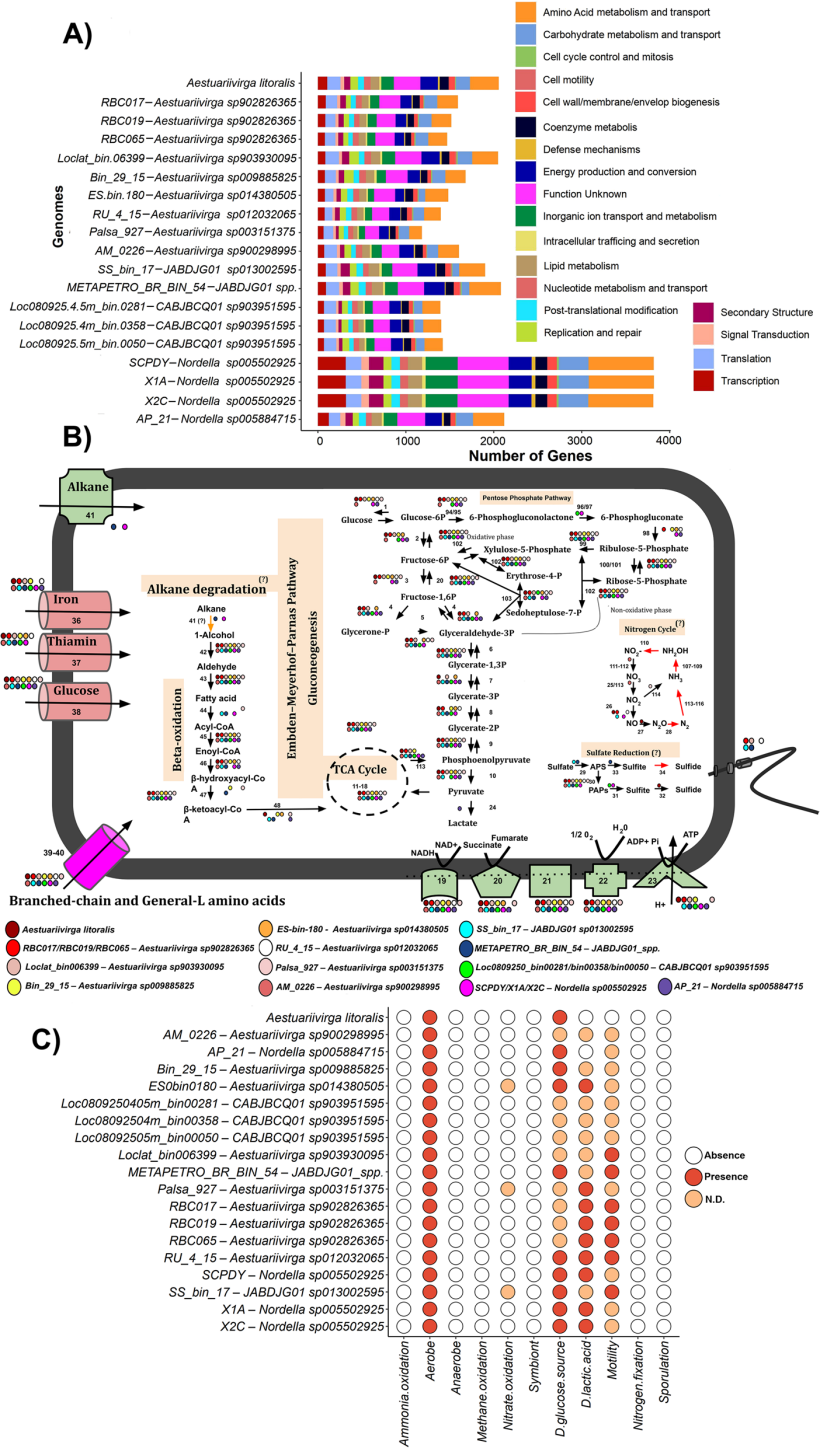
Functional profile of the Aestuariivirgaceae (Rhizobiales; Proteobacteria) family. **A** Abundance of general functions in each individual Aestuariivirgaceae genome. **B** Central metabolism of the Aestuariivirgaceae members. The model indicates the major putative functional predictions of the key pathways of Aestuariivirgaceae genomes. The pathways are highlighted by a pink colour and the question mark (?) symbol indicates incomplete pathways. A complete list of genes encoded by Aestuariivirgaceae genomes can be found in the Supplementary Table 2. Abbreviations: TCA, tricarboxylic acid cycle; ATP, Adenosine triphosphate. **C** Phenotype traits predicted by machine learning inferences

We found functional traits that may be useful in the ecological niche preferences of Aestuariivirgaceae (Fig. 2). Firstly, the most abundant general functions were associated with Amino Acid Metabolism and Transport, Functions Unknown, Energy Production and Conversion and Carbohydrate metabolism and transport (Fig. 2A). A similar pattern was observed in other Alphaproteobacteria members as described by Pini and collaborators [25]. As expected, many of these functions are also essential for central and accessory metabolism of Aestuariivirgaceae (Fig. 2B). The production of pyruvate from glucose uptake via the Embden-Meyerhof-Parnas (Glycolysis) pathway appeared to be a general trait of the Aestuariivirgaceae members. In addition, we also do not discard a possibility to also use Pentose Phosphate pathway as alternative via to uptake sugars. Yang, Heath & Setubal [26] pointed out that the LCA of all Rhizobiales showed any genes associated with Glycolysis/Gluconeogenesis. In this case, Aestuariivirgaceae metabolism would reflect a basal metabolism of Rhizobiales. The presence of Embden-Meyerhof-Parnas (Glycolysis) pathway also suggest that Aestuariivirgaceae family is well adapted to survive in environments rich in organic matter, as such marine sediments, soils [10, 12], estuarine ecosystems [16] and rivers [18], where the organic matter derived from biological biomass is abundant. Furthermore, *Nordella sp005884715* (AP_21 genome) has potential to perform pyruvate fermentation to lactate generation, which would represent adaptation and alternative metabolism to survive in soils (Fig. 2B). Machine learning predictions revealed with a high-confidence (> 0.7) the presence of D-glucose uptake (Fig. 2C) in ten species, corroborating our previous prediction analysing “gene-by-gene” in the metabolic reconstruction. We also infer that *Aestuariivirga litoralis* may living associated with particulate carbon in estuarine ecosystems, where organic matter degradation could continue via Embden-Meyerhof-Parnas (Glycolysis), but we also have not discarded its occurrence in a free-living water column. The same seems to be probably in the other *Aestuariivirga*, *Nordella* and *JABDJG01* and *CABJBCQ01* species described here, and reconstructed from soils, rivers, lakes and sediments, where organic matter is rich.

Still, regarding central metabolism and energy acquisition, member of the Aestuariivirgaceae family showed the main enzymes of the Electron Transport Chain and oxidative phosphorylation (Fig. 2B), including Ubiquinol-cytochrome c reductase cytochrome b/c1 (K00410) and Cytochrome c oxidase cbb3 (K00404), which are key-enzymes in the process to generate ATP using oxygen with final electron acceptor [27]. This result indicates that unlike other non-nitrogen-fixing Rhizobiales, such as Candidatus *Liberibacter asiaticus* and Candidatus *Liberibacter solanacearum* [28], the Aestuariivirgaceae genomes described here have the potential for aerobic respiration. As with glucose uptake metabolism, machine learning predictions also revealed with high-confidence (> 0.7) the presence of aerobic metabolism in all Aestuariivirgaceae investigated here (Fig. 2C). Furthermore, Li and collaborators [16] already validated experimentally this metabolic function in *Aestuariivirga litoralis*. Probably, many of the functional predictions described here may reflect the ecological role of these species in their environments, but it also needs experimental validations to better highlight all these predictions. Some new taxonomic groups were firstly described using assembly/binning approaches, and then in additional studies their putative functions were validated. The main recent example is the new archaea super-phylum Asgard archaea discovered in 2015 [29], where evolutionary and functional predictions were done by sequence analyses and 5 years later the first Asgard archaea Candidatus *Prometheoarchaeum syntrophicum* was cultivated [15].

Alternative metabolism to obtain energy could be present in Aestuariivirgaceae (Fig. 2B), but their presence is limited by homology unclear (I) or fragmented metabolic pathway predictions (II). The first case (I), which was related with homology unclear, was the presence of Alkane 1-monooxygenase (alkB - K00496) in *JABDJG01 spp*.(METAPETRO-BIN-54) and *Nordella sp005502925* (X2C, X1A and SCPDY) species. Both sequences showed a sequence identity of 45 and 40% respectively, and the presence of Alkane 1-monooxygenase (alkB - K00496) in both genomes could indicate a potential to use alkanes as growth substrates [30]. The presence of alkanes was not quantified in our sediment samples (METAPETRO-BIN-54) and also was not reported in the previous studies where the *Nordella sp005502925* (X2C, X1A and SCPDY) species genome were reconstructed [11]. Alternatively, regarding fragmented metabolic pathways (II), we also speculate that some Aestuariivirgaceae species could use a final electron acceptor derived from the nitrogen and sulfur cycles. We found an incomplete set of nitrogen cycle genes (por example, nitrite reductase/K00368/Denitrification and nitrate reductase/K00371/Nitrification), suggesting its potential to use nitrogen in respiration. In both cases shown here, we stressed that further studies are needed to investigate whether these functions are really active or only represent distant homologous genes or fragmented metabolic pathways.

Members of Aestuariivirgaceae showed an abundance of two-component proteins of OmpR family and response regulators of nitrogen (NtrC family) and cell cycle, contributing to the signal transduction process (Supplementary Table 2). Sec preprotein translocases seem to be a also useful mechanism for intracellular trafficking of majority bacterial Aestuariivirgaceae, with apparent general export pathway composed of a complex of SecD, SecE, SecF, SecG and SecY in the cytoplasmic membrane [31]. Furthermore, we also found genes of secretion and vesicular transport of effector molecules. As for the transference of genetic material between cell-to-cell interactions and T4SS enzymes, only *Aestuariivirga sp003151375* (Palsa_927) and *Nordella sp005502925* (SCPDY, X1A and X2X) showed potential to use bacterial conjugation (Supplementary Table 2). As for motility, *JABDJG01 sp*. (METAPETRO_BR_BIN_54), *Aestuariivirga sp903930095* (Loclat_bin-06399), *Aestuariivirga sp902826365* (RBC017, RBC019 and RBC065), *Aestuariivirga sp012032065* (RU_4_17) and *JABDJG01 sp013002595* (SS_bin_17) showed a functional flagella (Fig. 2B e 2C). Although the flagella absence has been reported for some Rhizobiales, we can infer that the Che and DviK proteins in the Aestuariivirgaceae family species can help circumvent a lack of motility [32, 33].

Finally, the absence of general phenotype traits associated with nitrogen fixation (e.g., nitrogeneses - *nif*) and pathogenicity (*virB/D*) (Supplementary Table 2), which is present in many Rhizobiales, would suggest a free-living lifestyle in the Aestuariivirgaceae members. This hypothesis agrees with previous results described by Wang and collaborators [34], which showed Rhizobiales has an ancient origin (~ 1500 Mya), and the last common ancestor of this order indicates that the free-living lifestyle was the base of their evolutionary trajectory. The phylogenetic relationship of Aestuariivirgaceae with free-living bacteria (such as *Hyphomicrobium*) observed in this study, leads us to hypothesize that the family members described here are probable free-living bacteria.

## Conclusion

In this study, we validate the monophyly of the Aestuariivirgaceae (Rhizobiales) family using phylogenomic methods, suggesting a basal split early taxonomic group. Together with functional annotation, we hypothesized that the presence of specific pathways (e.g., Glycolysis/Gluconeogenesis) and the absence of pathogenic genes in Aestuariivirgaceae could indicate a free-living lifestyle, similar to the Last Common Ancestor (LCA) of all Rhizobiales. These findings also reveal the presence of a versatile metabolism, from sugar degradation to hydrocarbon bioremediation, that allows these microorganisms to survive in the most diverse microbiomes, including soil and groundwater systems. Lastly, additional studies based on metatranscriptomics in environmental samples and culturomics of new Aestuariivirgaceae members will be necessary to identify and quantify gene functions predicted here.

## Methods

### Sequencing and assembly of marine sediment metagenomes

The total DNA from 28 marine sediment samples (0–2 cm (2,5 a 37 m) depth) collected across Brazilian southwest islands was extracted using the Quick-DNA Miniprep Kit (Zimo Research). Metagenomics libraries were constructed using the Nextera DNA Flex Library Prep Kit (Illumina) according to the manufacturer’s protocol. Sequencing was performed on an Illumina NextSeq 500 platform (2 X 150 bp) (Illumina, San Diego, CA) at Computational Genomics Unity Darcy Fontoura de Almeida (UGCDFA) of the National Laboratory of Scientific Computation (LNCC) (Petrópolis, RJ, Brazil). The marine sediment metagenomes were used to assemble genomes from metagenomes (MAGs) following these steps: Firstly, the Trimmomatic [35] was used to remove sequencing adapters and low-quality reads. Then, reads were assembled using Megahit [36]. Only contigs greater than 2500 bp were used in the binning step using Metabat2 [37]. To check the quality control of each individual potential genome (MAGs), we used the CheckM software [17] to estimate the completeness and contamination metrics. To estimate the taxonomy identification, we used the GTDB-tk software [38]. We used only MAGs with medium-quality draft (Completeness ≥50.0 and Contamination ≤5.0%) [14] in the taxonomic assignment.

### Aestuariivirgaceae (Rhizobiales; Proteobacteria) genomes available in the public database

All microbial genomes assigned as Aestuariivirgaceae family were retrieved from the Genome Taxonomy Database (GTDB) (July 2021) [9]. To selected and build an representative dataset with good quality genomes, we follow these criteria: firstly, we selected all genomes presenting a medium-quality draft (Completeness ≥50.0 and Contamination ≤5.0%) based on the Minimum information about a single amplified genome (MISAG) standards [14].

### Phylogenomic analysis

To estimate the phylogenetic position of the Aestuariivirgaceae family into the Rhizobiales order, we used a phylogenomic approach based on the alignment concatenation of 139 bacterial single-copy core genes (SCGs) (Supplementary Table 3) [39, 40]. Nineteen Aestuariivirgaceae genomes were used (Table 1) plus 39 Rhizobiales genomes and three other Bacteria (*Coraliomargarita akajimensis*, *Acidobacterium capsulatum* and *Escherichia coli*, which were used as outgroup). Each single-copy gene marker was identified using the HMM database from Campbell and collaborators [39] in Anvi’o software [40]. Each protein dataset was aligned using Muscle [41]. We excluded ambiguously aligned regions (−gt = 0.50) using trimAl v1.2 [42]. The alignments were concatenated to estimate the phylogeny using the JTT + CAT model in FastTree 2.0 software [43].

### Functional genome annotation

Each genome was annotated using an automated annotation workflow (SABIA) [44] to identify the open reading frame (ORF) and assign all functions based on the fast orthology assignment and precomputed eggNOG v5.0 clusters implemented in the eggNOG-mapper [45]. COG Functional Categories were used to summarize general functions and KEGG KO was used to investigate the main metabolic pathways. Machine learning inferences were used to predict the phenotype traits of each individual genomes using PhenDB [46].

## Supplementary Information

**Additional file 1 **: **Supplementary Table 1**. General informations about the metagenomic dataset used in this study to reconstruct METAPETRO_BR_BIN_54 genome. **Supplementary Table 2.** General features of metabolic pathways. **Supplementary Table 3**. Bacterial single-copy core genes (SCGs) from Campbell et al. [39] used in the phylogenomic analysis. (From Campbell et al. [39]).

## Data Availability

Metagenome-assembled genome (MAG) METAPETRO-BIN-54 was deposited in DDBJ/ENA/GenBank under the accession JAEKFU000000000. The version described in this paper is version JAEKFU010000000. Additional Aestuariivirgaceae genomes were deposited in public sequence repositories (GCF_003234965.1, GCA_003151375.1, GCF_005502925.1, GCF_005502975.1, GCF_005502345.1, GCA_005884715.1, GCA_009885825.1, GCA_014380505.1, GCA_012032065.1, GCA_013002595.1, GCA_900298995.1, GCA_903930095.1, GCA_903944365.1, GCA_903951595.1, GCA_903958745.1, GCA_902826365.1, GCA_902826675.1 and GCA_902826905.1).
